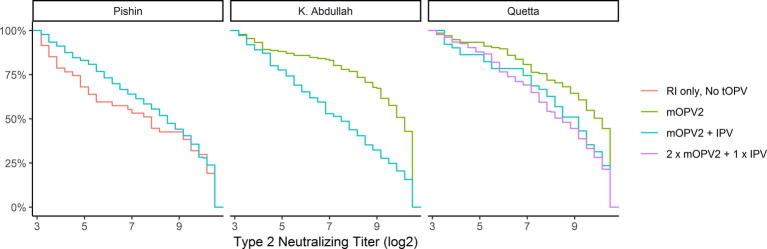# Immunity and field efficacy of type 2-containing polio vaccines after cessation of trivalent oral polio vaccine: A population-based serological study in Pakistan

**DOI:** 10.1016/j.jvacx.2020.100067

**Published:** 2020-05-06

**Authors:** Arie Voorman, Muhammad Atif Habib, Imtiaz Hussain, Rana Muhammad Safdar, Jamal A. Ahmed, William C. Weldon, Imran Ahmed, Muhammad Umer, Jeffrey Partridge, Sajid Bashir Soofi

**Affiliations:** a Department of Pediatrics & Child Health, The Aga Khan University, Pakistan; b Polio Program, Global Development, Bill & Melinda Gates Foundation, USA; c Polio Eradication Program, World Health Organization, Pakistan; d Polio National Emergency Operations Center, Govt of Pakistan, Pakistan; e Division of Viral Diseases, Centers for Disease Control and Prevention, Atlanta, USA

## Abstract

**Background:**

In Pakistan and other countries using oral polio vaccine (OPV), immunity to type 2 poliovirus is now maintained by a single dose of inactivated polio vaccine (IPV) in routine immunization, supplemented in outbreak settings by monovalent OPV type 2 (mOPV2) and IPV. While well-studied in clinical trials, population protection against poliovirus type 2 achieved in routine and outbreak settings is generally unknown.

**Methods:**

We conducted two phases of a population-based serological survey of 7940 children aged 6–11 months old, between November 2016 and October 2017 from 13 polio high-risk locations in Pakistan.

**Results:**

Type 2 seroprevalence was 50% among children born after trivalent OPV (tOPV) withdrawal (April 2016), with heterogeneity across survey areas. Supplementary immunization activities (SIAs) with mOPV2 followed by IPV improved population immunity, varying from 89% in Pishin to 64% in Killa Abdullah, with little observed marginal benefit of subsequent campaigns. In the other high-risk districts surveyed, a single SIA with IPV was conducted and appeared to improve immunity to 57% in Karachi to 84% in Khyber.

**Conclusions:**

Our study documents declining population immunity following trivalent OPV withdrawal in Pakistan, and wide heterogeneity in the population impact of supplementary immunization campaigns. Differences between areas, attributable to vaccination campaign coverage, were far more important for type 2 humoral immunity than the number of vaccination campaigns or vaccines used. This emphasizes the importance of immunization campaign coverage for type 2 outbreak response in the final stages of polio eradication. Given the declining type 2 immunity in new birth cohorts it is also recommended that 2 or more doses of IPV should be introduced in the routine immunization program of Pakistan.

## 1 Introduction

The use of Trivalent Oral Polio Vaccine (tOPV) has been successfully used to eradicate the three serotypes of polio (Type 1, 2 and 3) globally with the exception of Pakistan, Afghanistan and Nigeria [1], [2]. The tOPV has been the vaccine of choice in polio eradication due to its low cost and ease of use [3]. Presently wild type 1 serotype of polio is circulating in Pakistan where as wild type 2 has been eradicated globally and since 2012 wild type 3 has not been detected [2], [4]. Despite its effectiveness the tOPV is genetically unstable and has a tendency to be converted into a Sabin Like virus and the circulating vaccine-derived polioviruses (cVDPV) consequently causing the vaccine-associated paralytic poliomyelitis (VAPP) and outbreaks of cVDPVs in areas where immunization rates are low [3], [5], [6]. Among all three serotypes the type 2 component of tOPV has caused majority of VDPV and VAPP cases [4]. As wild type 2 poliovirus has been eradicated, the type 2 component of OPV was withdrawn from use globally in April 2016 (OPV2 cessation) to eliminate the burden of type 2 VAPP and cVDPV2 [7]. This switch from tOPV to bivalent OPV (bOPV) will significantly reduce the burden of VAPP, but Sabin Like 2 that was circulating prior to cessation could continue to circulate and lead to outbreaks of cVDPV2, further the risk of type 2 disease persists due to the sporadic emergence of type 2 circulating vaccine-derived poliovirus (cVDPV2), containment breaches, and persistent shedding from type 2-infected immunocompromised individuals in rare events [8], [9]. An OPV2 response will be required if such outbreaks occur [10]. After April 2016, monovalent OPV2 (mOPV2) is only intended to be used in response to cVDPV2 outbreaks, with bOPV substituted for tOPV in routine immunization with the addition of inactivated polio vaccine (IPV) [10].

Since IPV does not provide primary mucosal immunity, outbreaks caused by type 2 poliovirus require the use of monovalent type 2 oral polio vaccine (mOPV2) [11]. While mOPV2 is necessary to stop an outbreak, there is a risk that live Sabin 2 virus in the vaccine will circulate and eventually cause additional outbreaks of cVDPV2 [12], [13]. This risk increases if outbreak response campaigns are not of sufficient quality (coverage) or quantity. Under the assumed high potency of mOPV2, current outbreak response guidelines recommend two mOPV2 campaigns in target areas [14], [15]. However, there is a need to better understand the actual impact of mOPV2 campaigns to inform outbreak response policy. On the contrary IPV does not provide primary mucosal immunity, it can boost mucosal immunity among those who have previously received OPV [16], [17]. IPV can also limit pharyngeal shedding, which is believed to be a minor contributor to transmission [11]. However, IPV is more difficult to deliver than OPV, and high coverage is difficult to ensure. Currently, there is a global IPV shortage, and understanding the benefit of IPV in outbreak response campaigns is important to determine how it should be prioritized for that purpose.

In this paper, we present results from a sequential serological survey of children aged 6–11 months in Pakistan, conducted between November 2016 and October 2017. Studying this age cohort allowed us to assess the change in type 2 population immunity after tOPV cessation by comparing those born before and after cessation i.e. April 2016. In addition, supplementary immunization activities (SIAs) containing type 2 vaccine were also carried out during the surveys, allowing us to assess their impact by comparing children sampled before and after these campaigns. Notably, an outbreak of cVDPV2 was detected in Quetta district on 20 October 2016, which prompted a mOPV2 outbreak response between 2 January and 23 March 2017, consisting of a single round in all of Balochistan province and two rounds in Quetta district, followed by a single IPV campaign in Quetta block (Quetta, Pishin, and Killa Abdullah districts). Additionally, multiple IPV campaigns were carried out elsewhere in the country in response to WPV1 circulation. By comparing children sampled before and after campaigns and comparing to areas without campaigns, our survey provides a unique assessment of the impact of mOPV2 and IPV on population seroprotection against type 2 poliovirus.

## 2 Methods

### 2.1 Survey methodology

A sequential cross-sectional serological survey was conducted by Aga Khan University (AKU) in partnership with the polio national and provincial emergency operations centers, with the primary goal of informing and assessing strategies for stopping poliovirus transmission. The survey was conducted in two phases in each of 12 geographic strata (3 in Karachi, 3 in Quetta block, 2 in Peshawar, 2 in Khyber Pakhtunkhwa (KP)/Federally Administered Tribal Areas (FATA), and 2 in Northern Sindh). These areas were chosen for having recent evidence of WPV1 circulation. We also collected data from Rawalpindi and Lahore areas which served as control in phase 1 and phase 2 of the survey respectively.

For each phase of the survey, sampling in each geographic stratum consisted of 25 clusters. Clusters were chosen using probability proportional to size (PPS) and the polio program’s lot quality assurance sampling (LQAS) sampling frame. Once clusters were identified, field teams conducted a household enumeration and randomly selected 12 households with a child in the target age group (6–11 months) for participation in the study. Refusal and locked households were not replaced. Cluster and household random selection were done separately using the same methodology for each survey phase.

Following consent from the caregiver, basic demographic information, vaccination history, and socio-economic status data were obtained for all children included in the survey, along with 2 ml of venous blood that was drawn by trained phlebotomists. Ethical approval was obtained from the ethical review committees of the AKU.

### 2.2 Laboratory methodology

After clotting and centrifugation, sera were separated, transferred into labeled sterile cryovials and immediately stored in a cold box with ice packs and transported to the nearest laboratory collection point of AKU for further transportation to the AKU Nutrition Research Laboratory in Karachi. In the laboratory, two aliquots were prepared, one for backup and one for transport to the Centers of Disease Control at Atlanta, USA for analysis by neutralization assay [18]. In our survey the seropositivity was defined as titer of poliovirus neutralizing antibody ≥1:8 [19].

### 2.3 Statistical methodology

The primary outcome was type 2 seropositivity. Overall seropositivity was summarized separately by geographic strata, survey phase and birth cohort. Survey data were augmented with data on the number of supplementary immunization activities (SIAs) for which each child would have been eligible. IPV SIAs targeted children 4–23 months of age, while bOPV SIAs targeted all children under 5 years of age. Children were excluded from analyses of IPV SIA impact if they were present during an IPV SIA but under 4 months old. To assess protection offered by routine immunization only, immunity was assessed among children born after tOPV cessation and who were not eligible for SIAs containing type 2 vaccine.

To estimate SIA impact, we summarized immunity by area and SIA eligibility. SIAs that were conducted within 14 days of the blood draw were also excluded, since an immune response would not be expected without prior immunity. Where possible, we estimated intention to treat (ITT) seroconversion of the SIA as $1-\left(1-x_{\mathrm{post}}\right)/\left(1-x_{\mathrm{pre}}\right)$ where $x_{\mathrm{pre}}$ and $x_{\mathrm{post}}$ are the seroprevalence among children in an area before and after an intervention, who were otherwise comparable in terms of other opportunities for immunization. This was estimated by log-linked binomial regression. This model is a straightforward binomial generalised linear model (GLM) and in addition to its use in the relative risk regression it is commonly used in the analysis of serial survey data where age and time specific prevalence may be used to estimate age and time specific incidence.

All analyses accounted for survey design. For simplicity of exposition, we combined the three geographic strata in Karachi into a single unit and the two geographic strata in Peshawar into a single unit. When analyses included children from multiple strata or survey phases, each stratum was given the same weight, despite varying population sizes. This provided inference on immunity that is ‘typical’ across areas, rather than population averages that would be overly influenced by high population areas like Karachi. All analyses were conducted using R software [20], [21].

## 3 Results

### 3.1 Sample population

Table 1a shows demographic characteristics for each study area. There were 7940 children from 626 primary sampling units. Data and sample collection was started in November 2016 and ended in September 2017. Data collection for a single phase in a single geographic area lasted a median of 37 days (range 8–151 days). Wealth quintile wise the survey areas were pretty similar as the distribution of the population in the lowest wealth quintile was found to be about 20% in all survey areas, however the illiteracy rate of the respondents varied from 19% in Rawalpindi to 100% in Killa Abdullah. The percentage of male children ranged from 46% (Larkana) to 63% (Khyber). Immunization cards were available from > 50% of the caregivers in all target locations except Killa Abdullah, Larkana and Sukkur districts. Similarly, routine immunization with 3 doses of OPV (OPV3) was lowest (34%) in Killa Abdullah and highest (96%) in Lahore.

**Table 1a:** Basic demographic characteristics for each study area.

Area	Survey Phase	N	Clusters	SES (lowest Quintile %)	Education Level (Illiteracy rate- %)	Male (%)	Vaccination Card (%)	OPV3 Coverage (%)	IPV Coverage (%)
Karachi	1	1003	75	21.5	38.8	52	73	73	79
	2	971	75	21.6	33.7	53	84	68	74
Quetta	1	311	25	20.3	83.9	50	66	95	63
	2	317	25	20.1	91.5	51	50	54	61
Killa Abdullah	1	312	26	20.2	98.7	50	34	55	26
	2	326	25	20.2	100.0	52	40	34	40
Pishin	1	309	24	20.1	89.6	47	56	92	60
	2	310	25	20.0	96.5	50	50	45	50
Peshawar	1	627	50	20.4	80.9	53	85	77	83
	2	651	50	20.1	82.6	49	91	84	89
Khyber	1	301	25	20.2	95.4	63	73	73	63
Mardan & Swabi	1	314	25	20.1	63.0	56	86	90	94
	2	309	25	20.1	68.0	51	57	89	86
Larkana	1	341	25	20.2	88.6	46	43	58	66
	2	313	25	20.1	80.3	48	54	90	87
Sukkur	1	300	25	20.0	75.0	51	45	44	62
	2	298	25	20.1	94.3	54	34	36	56
Rawalpindi (Control)	1	327	26	20.1	19.2	52	93	95	98
Lahore (Control)	2	299	25	20.1	26.0	54	90	96	97

Table 1b shows the survey timing, timing of birth and eligibility of IPV and mOPV SIA in each study area. During the survey we identified children who were born before the switch, the data showed that 51% children in Karachi, 40% in Quetta, 52% in Pishin, 62% in Peshawar, 48% in Larkana, 53% in Sukkur and 27% in Rawalpindi were born before the switch. We also found that survey areas in Quetta, Killa Abdullah and Pishin were largely eligible for mOPV2 SIA.

**Table 1b:** Survey timing, timing of birth, IPV SIA and mOPV eligibility for each study area.

Area	Survey Phase	Blood Drawn		Birth Cohorts		Born before switch (%)	IPV SIA Eligibility (%)	mOPV2 SIA Eligibility (%)
Karachi	1	Nov-16	Mar-17	Dec-15	Sep-16	51	35	0
	2	Apr-17	Sep-17	May-16	Mar-17	0	48	0
Quetta	1	Jan-17	Feb-17	Feb-16	Aug-16	40	0	98
	2	Jul-17	Jul-17	Aug-16	Jan-17	4	81	100
Killa Abdullah	1	Mar-17	Mar-17	Apr-16	Sep-16	0	0	100
	2	Aug-17	Aug-17	Sep-16	Feb-17	0	72	95
Pishin	1	Dec-16	Jan-17	Jan-16	Jul-16	52	0	1
	2	May-17	Jul-17	Jun-16	Jan-17	0	94	100
Peshawar	1	Dec-16	Feb-17	Dec-15	Aug-16	62	8	0%
	2	Apr-17	Jul-17	Apr-16	Jan-17	0	70	0%
Khyber	1	Sep-17	Sep-17	Sep-16	Mar-17	0	17	0%
Mardan & Swabi	1	May-17	May-17	May-16	Nov-16	0	76	0%
	2	Oct-17	Oct-17	Oct-16	Apr-17	0	0	0%
Larkana	1	Jan-17	Feb-17	Jan-16	Aug-16	48	0	0%
	2	Aug-17	Aug-17	Aug-16	Feb-17	0	32	0%
Sukkur	1	Dec-16	Jan-17	Dec-15	Jul-16	53	0	0%
	2	Apr-17	May-17	May-16	Nov-16	0	75	0%
Rawalpindi (Control)	1	Mar-17	Mar-17	Mar-16	Sep-16	27	0	0%
Lahore (Control)	2	May-17	Jul-17	Jun-16	Dec-16	0	0	0%

### 3.2 Overall seroprevalence

**Fig. 1:**
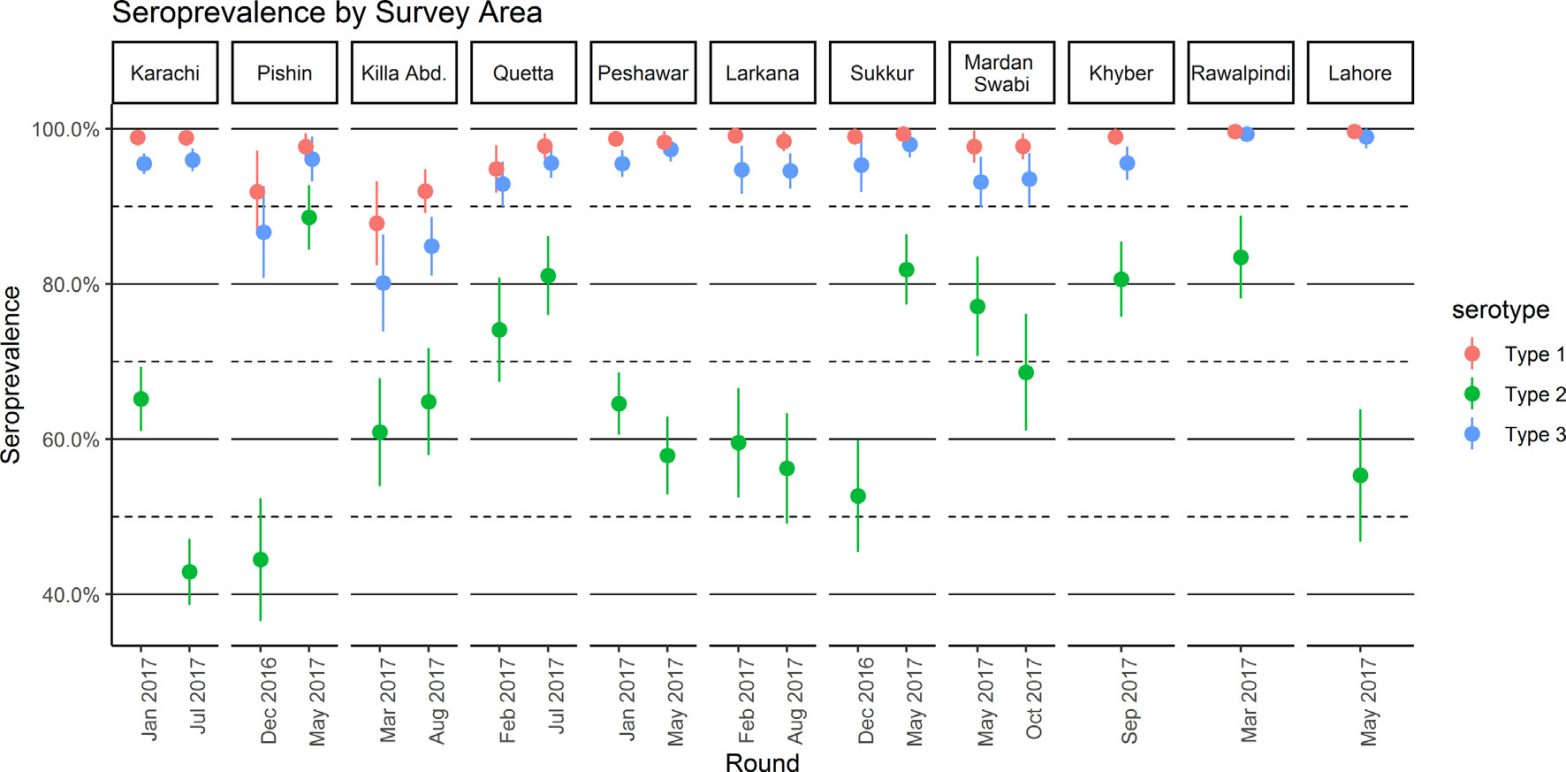
Seroprevalence for each survey area. Points represent estimates of seroprevalence, and lines indicate 95% confidence intervals. Dates on the x-axis indicate the median month of the blood draw for children in the survey round.

Immunity to serotypes 1 and 3 were > 90% for both phases and across geographic strata, with the exceptions found in Pishin and Killa Abdullah (Fig. 1). In contrast, type 2 seroprevalence was generally lower and more variable, ranging from 42% in Karachi in July 2017 to 89% in Pishin in May 2017. The large variation in type 2 immunity was due to a combination of the change from three doses of tOPV to one dose of IPV in routine vaccination following the tOPV cessation in 2016, variation in routine immunization coverage across geographic strata, and differing opportunities to receive supplementary type 2-containing vaccine across geographic strata and birth cohorts.

**Fig. 2:**
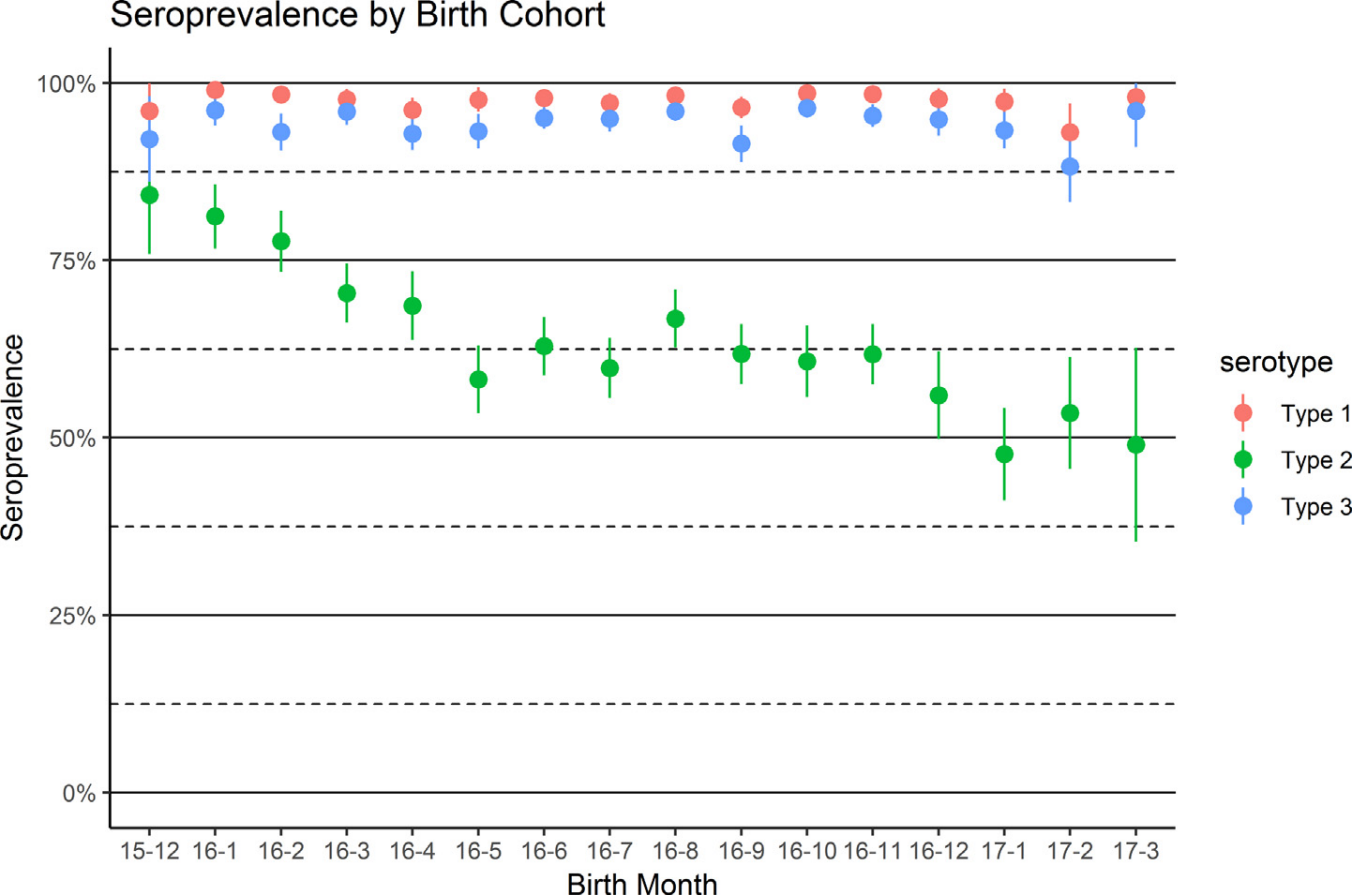
Seroprevalence by birth cohort. Points represent seroprevalence estimates, and lines indicate 95% confidence intervals. Dates on the x-axis indicate birth month in year-month format.

The overall type 2 seroprevalence declined in successive birth cohorts relative to types 1 and 3 (Fig. 2). Those born in December 2015 were eligible for tOPV in routine immunization in addition to tOPV SIAs conducted immediately prior to the vaccine switch, resulting in about 84% type 2 seroprevalence. In contrast, only about half of those born in 2017 were seropositive for type 2. For all birth cohorts, seropositivity for types 1 and 3 was consistently higher.

### 3.3 Effectiveness of IPV through routine immunization

**Fig. 3:**
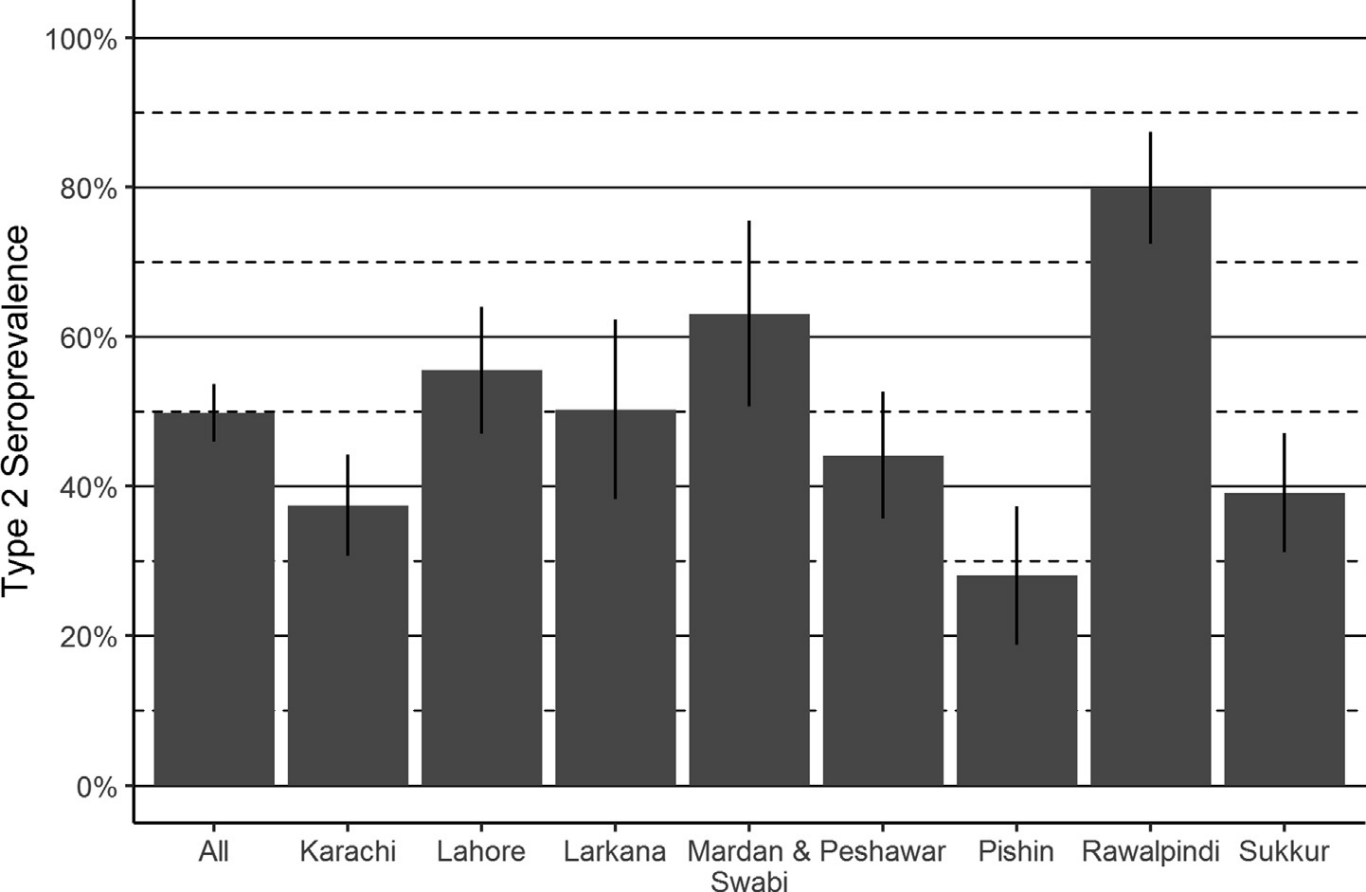
Type 2 seroprevalence through routine immunization, by study area. The heights of the bars represent seroprevalence while lines represent 95% confidence intervals.

To understand the protection offered through routine immunization, seroprevalence was estimated among those who were born after tOPV cessation and ineligible for mOPV2 or IPV SIAs. Among these 1534 children, 50% were seropositive for type 2, ranging from 28% in Pishin to 80% in Rawalpindi (Fig. 3).

### 3.4 Effectiveness of supplementary mOPV2 campaigns

**Fig. 4:**
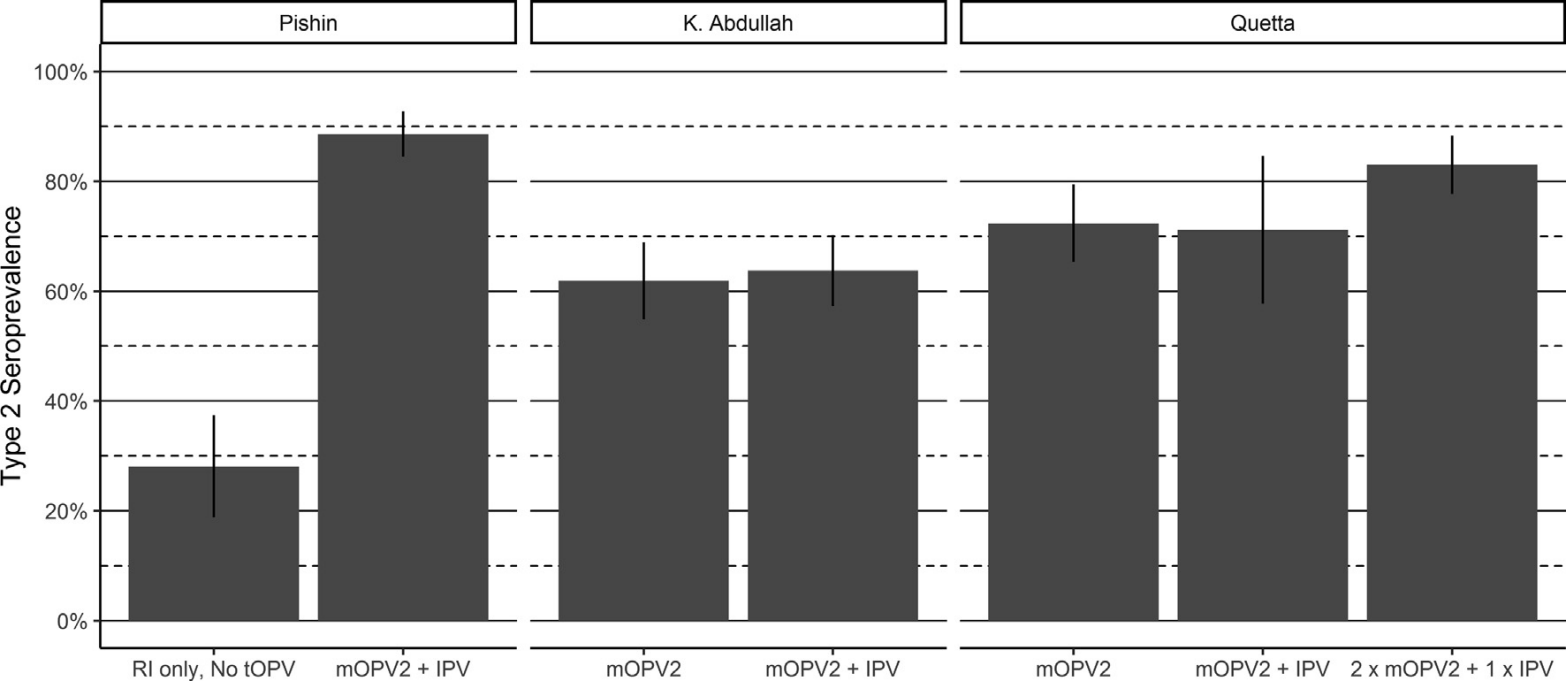
Seroprevalence among areas targeted with mOPV2. Bar height corresponds to seroprevalence, while lines show 95% confidence intervals.

**Fig. 5:**
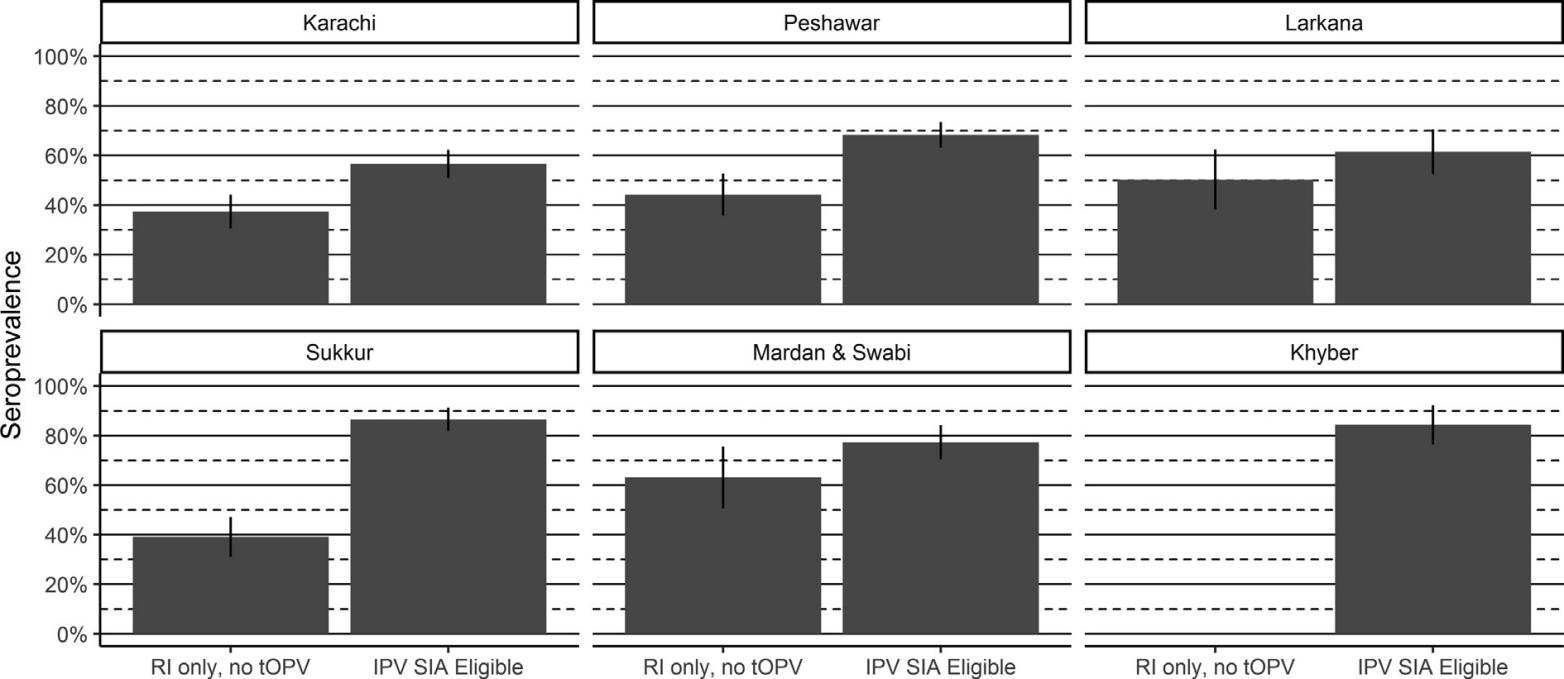
Type 2 immunity by IPV SIA eligibility. Bar height corresponds to seroprevalence, while lines show 95% confidence intervals.

**Supplementary figure 1:**
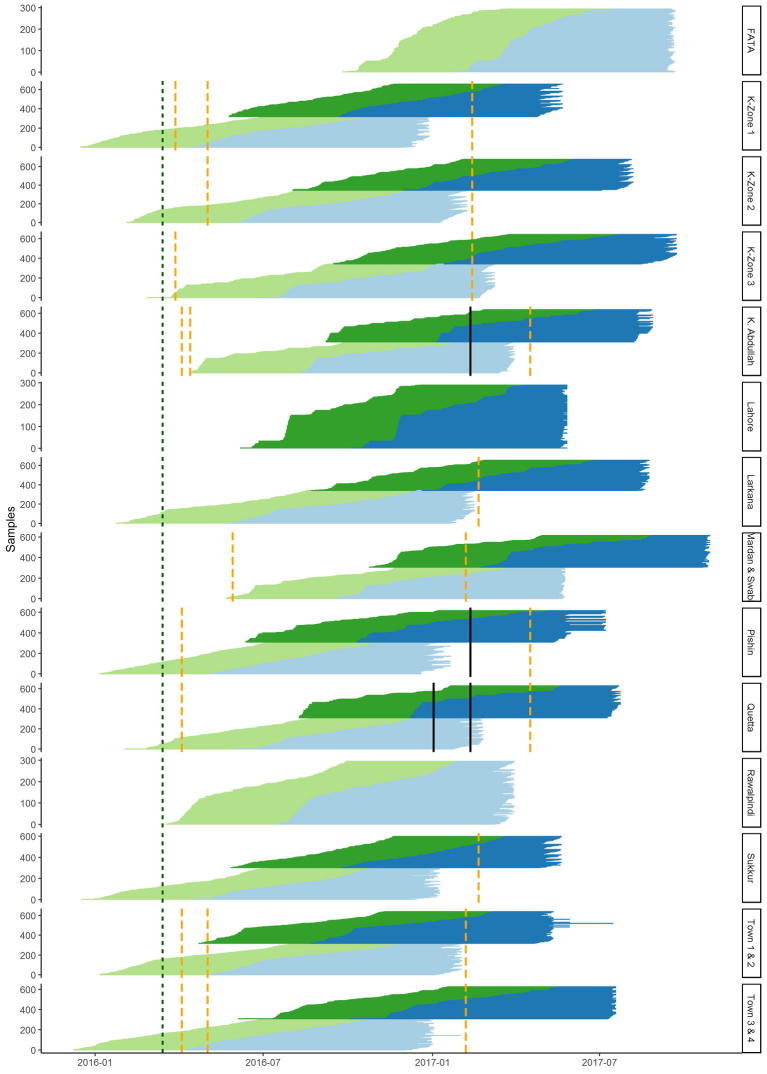


The cVDPV2 outbreak response campaigns in Balochistan were interspersed between serosurvey rounds, allowing evaluation of the effect of those campaigns on population immunity. The impact was seen most clearly in Pishin district, where the first round of the serosurvey included many children who were born after tOPV cessation and sampled before the outbreak response campaigns. Among children whose only opportunity for type 2 immunization was through routine immunization with IPV, type 2 seroprevalence was 28% (Fig. 4 and Table 2 Supplementary figure 1, Supplementary figure 2). Following the first survey round in Pishin, an mOPV2 SIA was carried out from 11 to 15 February 2017 in Pishin, Killa Abdullah and Quetta, followed by an IPV SIA from 17 to 23 April 2017 targeting the same districts. For the second survey round in Pishin district, carried out between May and July 2017, type 2 seroprevalence was 89% among those eligible for both SIAs, resulting in an ITT seroconversion of 83% for the combined mOPV2 + IPV SIAs (95% CI: 78%−88%).Fig. 5.

**Table 2:** Effectiveness of IPV and mOPV2 campaigns on type 2 seroprevalence.

		Reference Group		Comparison Group		
Area	Intervention	Vaccines	Seroprevalence (%) (95% CI)	Vaccines (in addition to reference)	Seroprevalence (%) (95% CI)	Intention-to- treat Seroconversion (%) (95% CI)
Lahore	Routine only	Routine only	56 (47, 64)			
Rawalpindi		Routine only	80 (72, 88)			
Karachi	IPV SIAs	Routine only	37 (31, 44)	IPV SIA	57 (51, 62)	31 (18, 41)
Peshawar		Routine only	44 (36, 53)	IPV SIA	68 (63, 73)	43 (29, 54)
Sukkur		Routine only	39 (31, 47)	IPV SIA	87 (82, 92)	78 (68, 85)
Larkana		Routine only	50 (38, 62)	IPV SIA	61 (52, 70)	22 (-9, 45)
Khyber		–		IPV SIA	84 (76, 92)	
Mardan & Swabi		Routine only	63 (51, 76)	IPV SIA	77 (70, 84)	38 (3, 61)
Pishin	mOPV2 and IPV SIAs	Routine only	28 (19, 37)	mOPV2 + IPV SIA	89 (84, 93)	84 (77, 89)
Killa Abdullah		mOPV2 SIA	62 (55, 69)	IPV SIA	64 (57, 70)	5 (–23, 27)
Quetta		mOPV2 SIA	72 (65, 79)	mOPV2 + IPV SIA	83 (78, 88)	39 (8, 59)

In neighboring Killa Abdullah district, the first round of the survey took place after the February mOPV2 SIA, but before the April IPV SIA. Type 2 seroprevalence was 62% among children eligible for the mOPV2 SIA (Fig. 4 and Table 2). For the second round in this district, conducted in August 2017, seroprevalence among those eligible for mOPV2 SIA and the additional IPV SIA was 64%. This resulted in an ITT seroconversion of 5% for the IPV SIA conducted after the mOPV2 SIA (95% CI: –23% − 27%).

In Quetta district, the first round of the serosurvey was conducted after a mOPV2 SIA was carried out from 2 to 6 January 2017. Blood was drawn largely during the subsequent mOPV2 SIA, too early for a serological response to be expected. Among these children, seroprevalence was 72% (Fig. 4 and Table 2). The second round of the survey in this district consisted of children who were eligible for two mOPV2 SIAs and a single IPV SIA, as well as a younger birth cohort that was born after January 2017, and only eligible for the second mOPV2 SIA and the subsequent IPV SIA. Type 2 seroprevalence was 71% for the younger mOPV2 + IPV group and 83% for the 2 × mOPV2 + 1 × IPV group. While power was limited to estimate an effect of the IPV campaign (ITT seroconversion estimate −3%, 95% CI: −73–39%), this suggests an ITT seroconversion of 39% (95% CI: 8–59%) for the combined mOPV2 and IPV SIAs.

### 3.5 Effectiveness of supplementary IPV campaigns

Outside of Balochistan, IPV campaigns were carried out in response to WPV1 outbreaks. The timing of the serological surveys gave an opportunity to assess their impact. In Karachi, the first round of serosurvey targeted children who were born after tOPV cessation and ineligible for IPV SIAs. Among this group, type 2 seropositivity was 37%, compared to 57% among those eligible for the IPV SIA, resulting in an ITT seroconversion of 31% (95% CI: 18–41%). A comparable trend was seen in Peshawar (43% seroconversion, 95% CI: 29–54%) with an increase from 44% to 68%.

Larkana and Sukkur had similar timings of the SIAs and survey rounds, but different trends in immunity among those eligible and not for IPV SIAs. In Larkana, the impact of the IPV SIA was not statistically significant (ITT seroconversion of 22%, 95% CI: −9–45%), while in Sukkur the SIAs improved immunity from 39% among those with routine immunization in the first survey round, to 87% among those in the subsequent survey round and who were eligible for an IPV SIA, compatible with an ITT seroconversion of 78% (95% CI: 68–85%).

In Khyber Pakhtunkhwa province, there was no reference group that had blood sampled before the IPV SIA. In both Mardan and Swabi districts, and in Khyber Agency, relatively high seroprevalence (approximately 80%) was seen among those eligible for an IPV SIA.

## 4 Discussion

Following tOPV cessation, a decline in protection against disease due to poliovirus type 2 was anticipated. We estimate that 50% of children in post-cessation birth cohorts in Pakistan are not protected against type 2 poliovirus, and as many as 72% of children in areas with low routine immunization coverage. As these new cohorts accumulate, it is likely that in the coming years the protection against type 2 will further decline. This supports the recommended two-dose IPV schedule, as well as efforts to improve routine immunization in Pakistan [22]. This declining immunity also highlights the increasing priority that should be given to ongoing and future type 2 outbreaks.

The timing of our study allowed for measurement of the impact of supplementary immunization activities using mOPV2 and IPV. We observed a strong response for the combined impact of mOPV2 and IPV SIAs in Pishin district, consistent with 84% ITT seroconversion, and resulting in 89% seropositivity among 6–11 months old children who were eligible for both SIAs. However, in neighboring Killa Abdullah district seroprevalence was only 64% among children eligible for the same mOPV2 and IPV SIAs, and 62% among those eligible for the mOPV2 SIA only. In Quetta district, an mOPV2 SIA alone resulted in 72% seroprevalence, while an additional mOPV2 SIA and IPV SIA improved immunity to 83%. This supports the findings in Voorman *et al.* 2017, who found that the first SIAs a cohort experience are likely the most beneficial, while subsequent SIAs have less impact [23]. As with that study, a likely explanation in the current study is coverage, where children who are not immunized in one SIA are highly likely to remain unimmunized through subsequent SIAs. However, when comparing with type 1 and type 3 immunity, which benefit from many more SIAs, we note that high immunity was eventually achieved in the same population.

Though the cVDPV2 outbreak in Balochistan has apparently been stopped, this study cannot assess what immunity level is an appropriate target for outbreak response. Our study involved children 6–11 months old, chosen as the most vulnerable age group. However, the contribution of older age groups with higher immunity and prior tOPV exposure was likely relevant to the apparent termination of the cVDPV2 outbreak in Quetta.

Our results show that IPV SIAs can have a substantial effect on type 2 seroprotection in settings of low immunity. The impact of IPV campaigns on transmission could not be determined, since the study did not assess viral shedding or mucosal immunity.

The study is limited by its observational nature. Cohorts and sample collection were not designed to evaluate routine immunization and SIA impact; as a result, while age is important for vaccine efficacy, the age at which an SIA occurred is not the same between comparison groups. Likewise, sources of immunity could not be cleanly distinguished, and there remain some survey areas for which there is no simple explanation for the observed data. Seroprevalence in Rawalpindi (80%) is greater than one would expect from a single dose of IPV that 85% of the children reported. Further, it contrasts with much lower protection in Lahore, where 70% of children reported three doses of IPV, likely from private clinics.

## 5 Conclusions

Type 2 immunity has declined among cohorts born after tOPV cessation in Pakistan and, thus, overall population protection against type 2 poliovirus will decline without both improvements in routine immunization and introduction of two or more doses of IPV. While risk of type 2 polio outbreaks is likely decreasing in Pakistan, the ability of an outbreak to spread rapidly is increasing.

Supplementary immunization campaigns with mOPV2 and/or IPV resulted in substantial improvements in seroprotection in some survey areas, but the effect was not uniform. The vaccine used in an SIA, whether mOPV2, IPV, or both, is not a guarantee of high population immunity to type 2 poliovirus. Further, the impact of subsequent SIAs beyond the first was lower in general, suggesting that children missed in one SIA are more likely to remain missed in subsequent SIAs.

## Declaration of Competing Interest

The authors declare that they have no known competing financial interests or personal relationships that could have appeared to influence the work reported in this paper.

## Figures and Tables

**Supplementary figure 2:**